# Hyperbranched Polysiloxanes Based on Polyhedral Oligomeric Silsesquioxane Cages with Ultra-High Molecular Weight and Structural Tuneability

**DOI:** 10.3390/polym10050496

**Published:** 2018-05-04

**Authors:** Ning Liu, Jianyi Yu, Yaoyong Meng, Yuzhou Liu

**Affiliations:** 1 MOE Key Laboratory of Laser Life Science & Laboratory of Photonic Chinese Medicine, College of Biophotonics, South China Normal University, Guangdong 510631, China; liuningmengqi@163.com; 2 Beijing Advanced Innovation Center for Biomedical Engineering, Beihang University, Beijing 100191, China; yujianyi9058@163.com; 3 School of Chemistry, Beihang University, Beijing 100191, China

**Keywords:** polyhedral oligomeric silsesquioxanes, high molecular weight, nanoparticles

## Abstract

Hyperbranched siloxane-based polymers with ultra-high molecular weight were synthesized by the Piers–Rubinsztajn reaction between octakis(dimethylsiloxy) octasilsesquioxane with different dialkoxysilanes, using tris(pentafluorophenyl) borane as the catalyst. The origin of the high molecular weight is explained by the high reactivity of the catalyst and strain energy of isolated small molecule in which all eight silane groups close into rings on the sides of a single cubic cage. The structural tuneability was further demonstrated by use of methyl(3-chloropropyl)diethoxysilane, which generates a polymer with similar ultra-high molecular weight. Introduction of phosphonate groups through the chloropropyl sites later leads to functionalized polymers which can encapsulate various transition metal nanoparticles.

## 1. Introduction

Polyhedral oligomeric silsesquioxanes (POSSs), as first synthesized by D.W. Scott in 1946 [1], have attracted considerable attention. POSSs are polysiloxanes consisting of the structure unit of (RSiO_3/2_)_n_, and they can exist in different forms, including amorphous, cage-type, dumbbell-shape, and ladder-structured ones [2,3,4,5,6,7,8,9]. Among them, cage-type POSSs have been investigated extensively, owing to their well-defined nano-sized structures. Polymers possessing POSS cages as structural subunits can have special properties, such as improved mechanical and thermal properties [10,11,12,13], and therefore the synthesis of such polymers has constantly been an attractive research direction.

POSS-cage-containing polymers can be designed and prepared in several different topologies depending on the synthesis methodologies, among which the controlled radical polymerization has received most attention. The alkene functional groups are first introduced into POSS cage molecules through organic synthesis, and then polymerization techniques such as RAFT [14,15,16,17], ATRP [18,19], ROMP [20,21,22] allow subsequent generation of POSS-cage-containing polymers either by themselves or with other alkene monomers. Other ways, such as coupling reactions, have also been utilized for a similar purpose. Depending on the number of functional groups within one POSS cage, the morphology of the resulting polymers can be radically different. The POSS cages with two connecting points can be incorporated into linear polymer backbones, and they can be attached sidelong of linear polymers if only one connecting point is present [23,24,25,26,27,28]. POSS cages with multiple connecting points usually lead to cross-linked polymers, and the special geometry of POSSs can make the polymers possess interesting permanent porosity [29,30,31]. 

However, for POSS cage monomers with multiple connecting points, it is also possible to generate soluble hyperbranched structures with POSS-cage incorporated either in the core or on the periphery with judicious design, and such polymers are of particular interest since they offer unique properties such as low viscosity and internal free space, not seen in other morphologies. Up to now, most hyperbranched POSS-cage-containing polymers have been prepared involving carbon-based chemistry in the backbone construction [32]. However, the presence of C–O or C–N bonds in the backbone can significantly deteriorate the thermal performance of such polymers even in the presence of POSS subunits. Polysiloxanes are special polymers which only involve strong Si–O bonds in the backbone, and therefore can exhibit much improved thermal performance. In this regard, of particular interest to us is the hyperbranched POSS-cage-containing polysiloxanes (HPCCPs), since it offers all-siloxane type cage polymers, which can be possibly used in harsh conditions.

HPCCPs have been prepared from octakis(dimethylsiloxy) octasilsesquioxane (Q_8_^DMS^), which reacts with themselves or with phenylsiloxanediols through dehydrogenative polycondensation under certain catalysts [33,34,35]. However, the insufficient catalysis efficiency in these reactions failed to produce polymers with high molecular weight. As the reaction goes, the concentration of reactive groups gradually drops to a level when active ends of low molecular weight (MW) polymers stop to react further and the polymer stop to grow. The MW is usually between 2 to 50 × 10^3^ dalton. In addition, the choice of starting materials is quite limited due to necessary use of unstable siloxanediols, resulting a poor structural variety in the final polymer. Therefore, there currently is a need to develop new methodologies to access high MW and rich structural tuneability in HPCCPs, which can endow more opportunities to such siloxane-based materials than what they have now.

We previously reported the synthesis of hyperbranched polysiloxanes through de-alkyl coupling reaction between hydrosilane and tetraethylorthosilicate catalyzed by B(C_6_F_5_)_3_ [36]. The high efficiency obtained encouraged us to investigate the possibility of combining Q_8_^DMS^ with different dialkoxysilanes to generate HPCCPs. The high catalysis efficiency can promise chances to achieve high MW polymers, while the redundant kinds of dialkoxysilanes available either commercially or through simple synthesis provide opportunities for structural variety.

Herein, we would like to report our work in this direction on the synthesis of HPCCP with ultra-high MW (>1 × 10^6^) by use of simple starting materials. The great size and available functional groups enabled core environment modification and subsequent encapsulation of transition metal nanoparticles within the polymer.

## 2. Experimental

### 2.1. Materials and Methods

All reactions were carried out under an air atmosphere in oven-dried single-neck round bottom flasks with magnetic stirring bars. Tris(pentafluorophenyl)borane, Q_8_^DMS^, cyclohexane, diphenyldimethoxysilane, 3-chloropropylmethyldiethoxysilane, triethyl phosphite, tetrahydrofuran, methanol, toluene, hexadecyltrimethylammonium chloride, sodium tetrahydroborate (NaBH_4_), silver nitrate (AgNO_3_), sodium chloropalladate (Na_2_PdCl_4_), and sodium tetrachloroaurate(III) dehydrate (NaAuCl_4_·2H_2_O) were purchased from InnoChem, Inc., Pudong, China, and then used without any further purification. Water was distilled before use. ^1^H-NMR (400 MHz) spectra were recorded in CDCl_3_ on a Bruker Avanced 400 NMR spectrometer. The ^1^H-NMR spectra were referenced to residual solvent signals at 7.26 ppm (CHCl_3_). ^29^Si-NMR spectra was recorded in CDCl_3_ with a 60 s delay at 120 MHz. Gel-permeation chromatography (GPC) analysis was carried out on a Viscotek GPC/SEC 270 max with RI/UV/viscosity/light scattering as the detectors, and toluene was used as the eluent. Dynamic light scattering (DLS) tests were carried out in THF with the concentration of 8 mg/mL on the Zetasizer Nano ZS90. For Transmission Electron Microscopy (TEM) imaging, the dichloromethane solution of relative samples (sodium chloropalladate/silver nitrate/sodium tetrachloroaurate(III) dehydrate) with the concentration of 0.001 mmoL/mL was drop-coated on the copper film, and were then imaged after drying in the air by HT7700 apparatus of HITACHI with the accelerating voltage of 200 kv. Thermogravimetric analysis (TGA) was conducted by TA SDT-Q600 either in the air or nitrogen at the heating speed of 10 °C/min with around 5 mg sample. Fourier Transform Infrared Spectroscopy (FTIR) was done by Nicolet 6700 FT-IR spectrometer on the KBr pallet with the transmission mode in the sample concentration range of 0.1–1.0 wt %. Single-crystal X-ray diffraction was conducted on Bruker Apex II single-crystal X-ray diffraction with Mo source.

### 2.2. Synthesis of Polymer ***1***

Tris(pentafluorophenyl)borane (B(C_6_F_5_)_3_) (20.5 mg, 0.04 mmol) was dissolved in 1 mL cyclohexane in a 10 mL two-neck flask equipped with a stirring bar. A mixture of Q_8_^DMS^ (0.509 g, 0.5 mmol) and diphenyldimethoxysilane (0.489 g, 2 mmol) in 6.25 mL cyclohexane was slowly added to the catalyst solution. After 1 h, the solvent was removed using rotovap to obtain a white solid powder which was then dissolved in a small amount of tetrahydrofuran and precipitated out using methanol. The product was then washed repeatedly with methanol. After washing, the solid was dried under an oil pump to give white solid powder (polymer **1**, 0.586 g, yield 62%).

### 2.3. Synthesis of the Crystal ***1***

B(C_6_F_5_)_3_ (40 mg, 0.0781 mmol) was dissolved in 50 mL cyclohexane at 50 °C in a 250 mL two-neck flask. A mixture of Q_8_^DMS^ (6.3625 g, 6.25 mmol) and diphenyldimethoxysilane (6.109 g, 25 mmol) in 40 mL cyclohexane was added to the catalyst solution. After addition, the reaction mixture was stirred for another 2 h. The reaction mixture was then filtered, and the filtrate (about 100 mL) was slowly added to MeOH (300 mL). The solution was then transferred to a refrigerator, and kept overnight. The crystals were then collected through filtration (crystal **1**, 0.44 g, yield 3.5%).

### 2.4. Synthesis of Polymer ***2***

B(C_6_F_5_)_3_ (20 mg, 0.04 mmol) was dissolved in 10 mL cyclohexane in a 50 mL two-neck flask equipped with a stirring bar. A mixture of Q_8_^DMS^ (0.508 g, 0.5 mmol) and 3-chloropropylmethyldiethoxysilane (0.42 g, 2 mmol) in 2.5 mL cyclohexane was slowly added to the catalyst solution. After 1.5 h, the solvent was removed using a rotovap to obtain a white solid powder which was then dissolved in a small amount of tetrahydrofuran and precipitated using methanol. The product was then washed repeatedly with methanol. After washing, the solid was dried under an oil pump as white powder (polymer **2**, 0.13 g, yield 16%).

### 2.5. Synthesis of Polymer ***3***

Synthesis of polymer **3**: polymer **2** (100 mg) and triethyl phosphite (0.16 g, 0.98 mmol) were mixed and heated at 180 °C. After 48 h, the reaction mixture was concentrated and dissolved in 1 mL tetrahydrofuran. Excessive MeOH was added to induce precipitation. After additional MeOH washing, the white solid was dried under an oil pump (polymer **3**, 0.025 g, yield 65%).

### 2.6. Transition Metal Nanoparticle Encapsulation by Polymer ***3***

Gold nanoparticle encapsulation: a solution of NaAuCl_4_ (0.95 mg, 0.0024 mmol) in deionized H_2_O (1 mL) was added to the solution of hexadecyltrimethylammonium chloride (3.84 mg, 0.012 mmol) in CH_2_Cl_2_ (1 mL). The resulting mixture was stirred until Au (III) was transferred into the organic phase (~60 min). A solution of polymer **3** (5 mg) in CH_2_Cl_2_ (1 mL) was added. Subsequently, the gold salts in the solution was reduced by addition of an aqueous solution of NaBH_4_ (2.7 mg, 0.072 mmol) in deionized H_2_O (1 mL). The resulting organic layer was in mauve. The CH_2_Cl_2_ layer was separated and used for transmission electron microscopy (TEM) analysis.

Silver nanoparticle encapsulation: a solution of AgNO_3_ (1 mg, 0.00388 mmol) in deionized H_2_O (1 mL) was added to the solution of hexadecyltrimethylammonium chloride (6.21 mg, 0.0194 mmol) in CH_2_Cl_2_ (1 mL). The resulting mixture was stirred until Ag (I) was transferred into organic phase (~60 min). A solution of polymer **3** (1.97 mg) in CH_2_Cl_2_ (1 mL) was added. Subsequently, the silver salts in the solution was reduced by addition of an aqueous solution of NaBH_4_ (4.4 mg, 0.1164 mmol) in deionized H_2_O (1 mL). The resulting organic layer was in earthy yellow. The CH_2_Cl_2_ layer was separated and used for TEM analysis.

Palladium nanoparticle encapsulation: a solution of Na_2_PdCl_4_ (0.59 mg, 0.002 mmol) in deionized H_2_O (1 mL) was added to the solution of hexadecyltrimethylammonium chloride (3.2 mg, 0.01 mmol) in CH_2_Cl_2_ (1 mL). The resulting mixture was stirred until Pd (II) was transferred into organic phase (~60 min). A solution of polymer **3** (1.01 mg) in CH_2_Cl_2_ (1 mL) was added. Subsequently the palladium salts in the solution was reduced by addition of an aqueous solution of NaBH_4_ (2.3 mg, 0.06 mmol) in deionized H_2_O (1 mL). The resulting organic layer was in black-brown. The CH_2_Cl_2_ layer was separated and used for TEM analysis.

## 3. Results and Discussion

### 3.1. Synthesis of Polymer ***1***

**Scheme 1:**
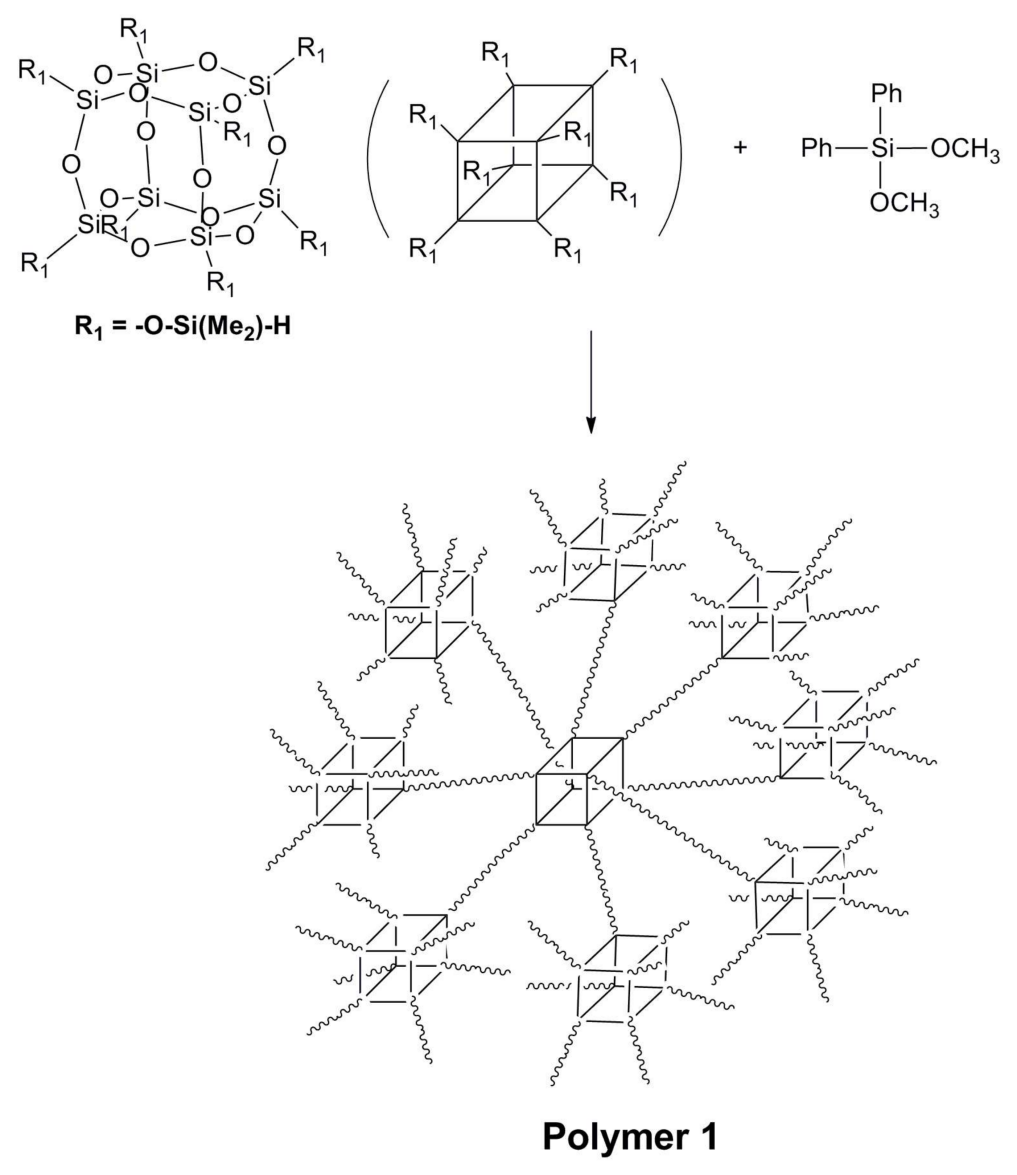
Reaction scheme to prepare polymer **1**.

As shown in Scheme 1, polymer **1** was synthesized by the reaction of Q_8_^DMS^ with diphenyldimethoxysilane in cyclohexane, using tris(pentafluorophenyl) borane as the catalyst. The reaction proceeded very fast, and upon addition of reactants into the catalyst solution, gas bubbles formed immediately, indicating the releasing of methane gas and formation of Si–O–Si bond. The observed high efficiency of so-called Piers–Rubinsztajn reaction is consistent with previous reports [37,38]. The obtained product after precipitation purification is a white solid and dissolves in common solvents such as THF, dichloromethane, and cyclohexane very well, but not in methanol. 

**Figure 1:**
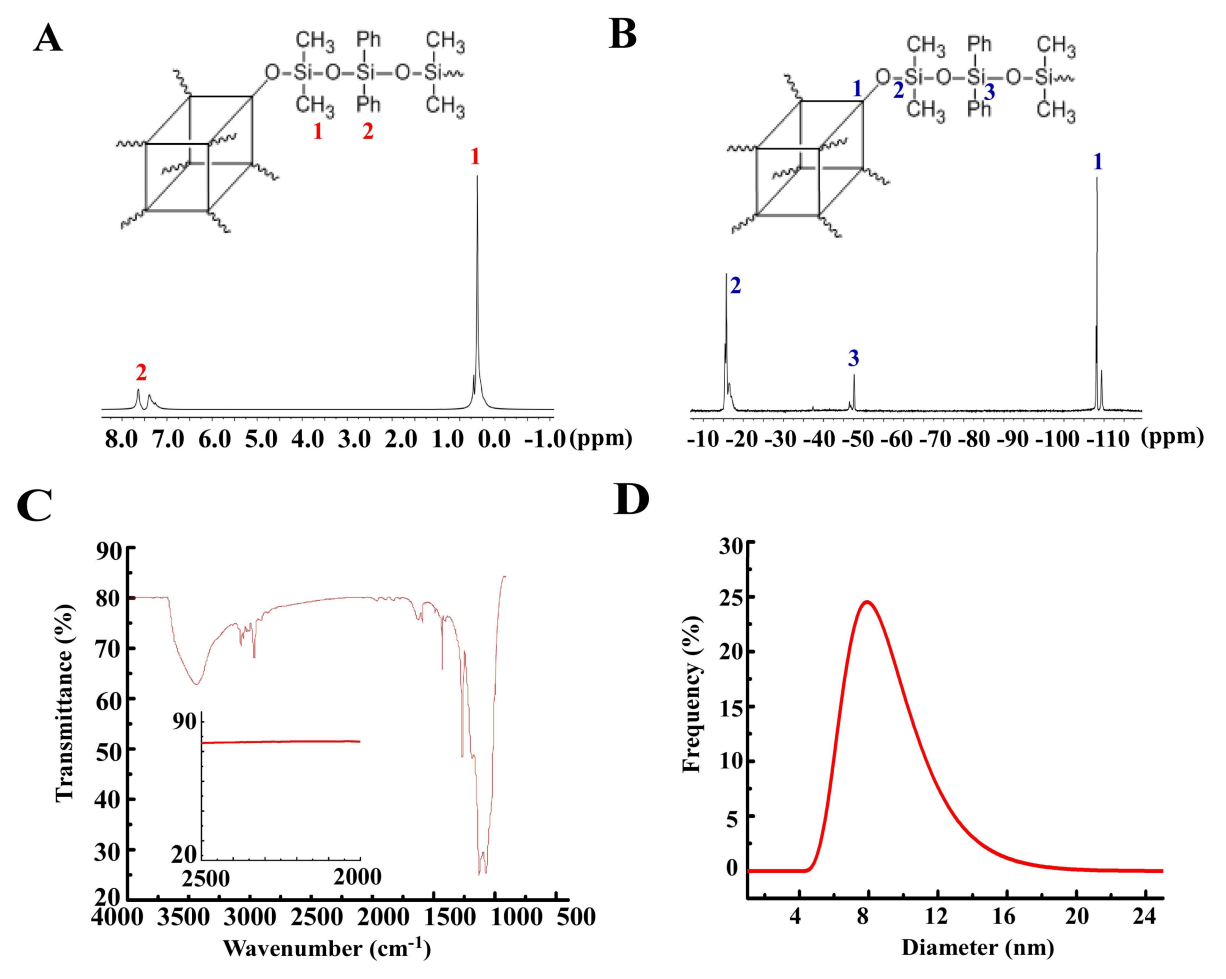
(**A**) the ^1^H-NMR of polymer **1** in CDCl_3_; (**B**) the ^29^Si-NMR of polymer **1** in CDCl_3_; The Q region was contaminated by the Q Si atoms in the NMR tube, and the spectrum was then flatten particularly in that region for the sake of clarity. (**C**) the IR of polymer **1** between 2000 and 2500 cm^−1^; (**D**) the dynamic light scattering (DLS) of polymer **1**, showing the diameter of around 8 nm.

The absolute molecular weight of polymer **1** was measured with GPC equipped with light scattering and viscosity detector. Surprisingly the MW was found to be as large as around 4.1 million (weight-averaged), corresponding to around 2200 POSS subunits. The polydispersity index (PDI) was around 1.2 (Figure S9 and Table S1). The giant size of the polymer is also confirmed by dynamic light scattering experiments, which indicates the averaged diameter of around 8 nm for a single molecule of polymer **1** (Figure 1D). The huge molecular weight is unprecedented for any POSS-cage containing polysiloxanes. Polymer **1** can be possibly regarded as one example of single molecular particles given its hyperbranched structures, and their exact solution behavior, whether adopting a fixed geometry and shape or randomly folded structures, remains as a curiosity which probably needs further investigation with respect to effect of incorporated rigid POSS cages.

We attributed the formation of high molecular weight polymer **1** to the high efficiency of the Piers–Rubinsztajn reaction, which forces the full consumption of the hydride and methoxy groups, and therefore the continuing growing of the polymer into a giant size. The IR spectrum of polymer **1** (Figure 1C) shows the absence of any peak between 2250 and 2100 cm^−1^, indicating the full consumption of Si–H groups in polymer **1**. The complete absence of terminating hydride is also confirmed by ^1^H/^29^Si-NMR in Figure 1A,B), which also indicates the absence of any methoxy leftover within polymer **1**, detailed analysis in Figures S3 and S4. These results are highly similar to our previous report about the reaction between tetramethyldisiloxane and tetraethylorthosilicate, in which all hydride and methoxy groups fully react to form a hyperbranched polymer [36].

In addition to the proposed hyperbranched structure as shown in Scheme 1, it is also reasonable to expect cross-linked particles to form during the reaction. However, the absence of precipitation after the reaction indicated high proclivity of the reaction to formation of the hyperbranched version instead of the cross-linked one. The full consumption of all reactive groups, as discussed above, combined with the good solubility of polymer **1**, strongly suggests the assumption that significant intramolecular cyclization reaction must happened at the later stage of reaction. As the reaction goes, the concentration of small molecular reactants will drop to a level where the intramolecular reaction overwhelms the intermolecular reaction, and the intramolecular cyclization will happen predominantly to close up the polymer, which is then inert for further growth. 

**Scheme 2:**
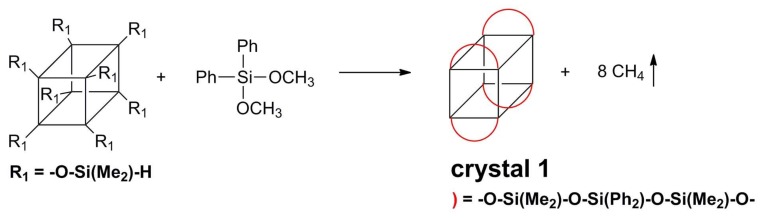
Reaction scheme of crystal **1**.

**Figure 2:**
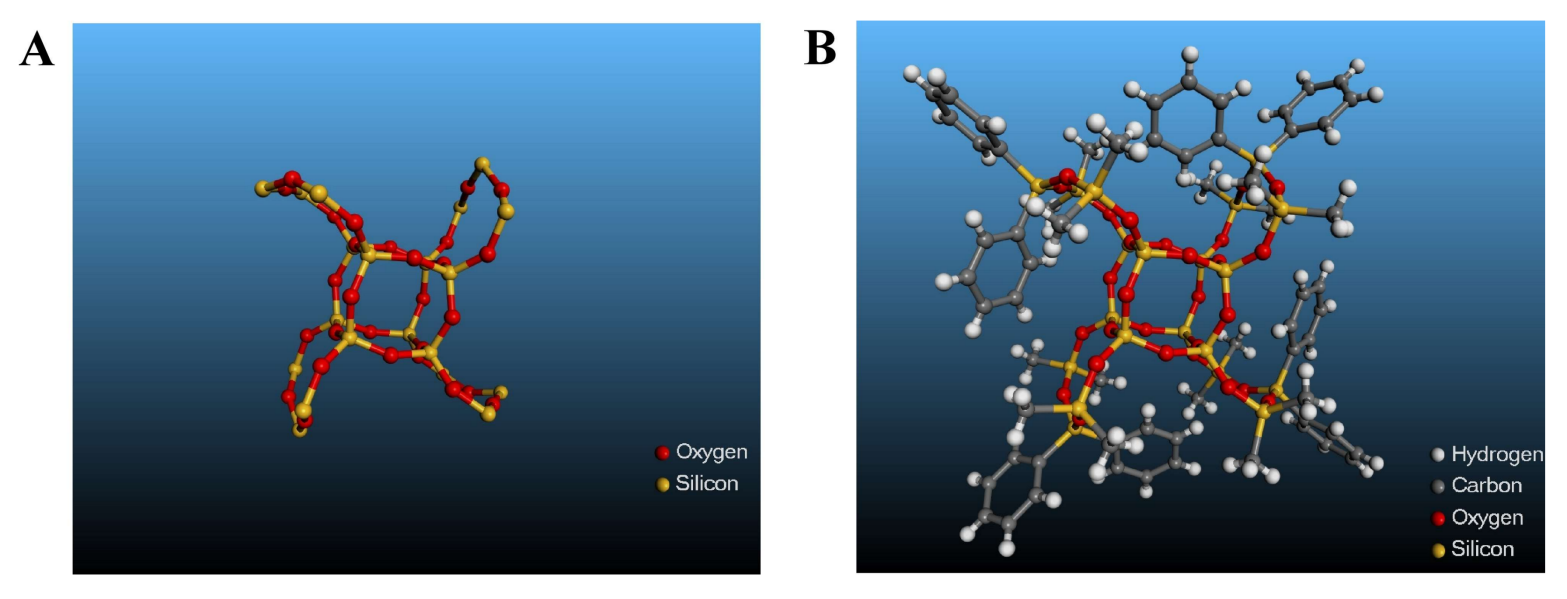
Images of the single crystal structure of crystal **1** without (**A**) and with (**B**) carbon and hydrogen elements.

In order to prove this assumption, we carried out the reaction in a dilute concentration in order to induce the intramolecular cyclization reaction to happen at a much earlier stage in the hope of obtaining well-characterized small molecule. Luckily, we successfully obtained the single crystal structure of smallest version of polymer **1** (crystal **1**, around 3.5%), in which all adjacent hydride groups react intramolecularly with diphenyldimethoxysilane to close into a loop on the side of the POSS cage, as shown in Scheme 2. This small molecule forms nice block-like crystal to permit us conduct single-crystal X-ray diffraction on it. The unit cell of crystal **1** belongs to the triclinic group, and there is one POSS molecule for each unit cell (See Table S2 for unit cell parameters). The relative ^1^H- and ^29^Si-NMR spectra (Figures S1 and S2) confirmed the structure. As shown in Figure 2, the full preservation of the POSS cage is consistent with the benign nature of the B(C_6_F_5_)_3_ catalyst towards the Si–O bonds, and the Si–O bonds are usually uninterrupted during the Pier–Rubinsztajn reaction as shown by Brook et al. [39]. The structural rearrangement through the cleavage and reformation of Si–O bonds by a lot of catalysts has been troublesome [29] and this problem seems to absent in our approach, therefore maximizing the structural fidelity of generated polymers according to the design rules.

The structural feature of crystal **1** sheds light into the structure of polymer **1**. As shown in Figure 2, the cyclopentasiloxane rings on the side of POSS cage in crystal **1** are all significantly distorted to one direction presumably due to the crowded surface of the POSS cage, and therefore the cyclopentasiloxane ring in crystal **1** should be more constrained than regular ones [40,41,42,43]. In other words, there will be lesser chance for the polymer to close up and stop growing due to the crowded space, and this facilitates the formation of high MW polymers. Only when polymer grows to a giant size, the intramolecular cyclization may happen due to the significantly reduced chance for intermolecular reaction with other reactive small molecules, and then the polymers stop growing. Combined with the absence of SiH or SiOMe groups, as supported by ^29^Si- and ^1^H-NMR, the end groups of polymer **1** are estimated to be mostly intramolecular loops. However it has to be pointed out that the possibility of other kind of intramolecular cyclization with polymer **1**, other than the one shown in Figure 2 cannot be completely ruled out. 

### 3.2. TGA of Polymer ***1***

**Figure 3:**
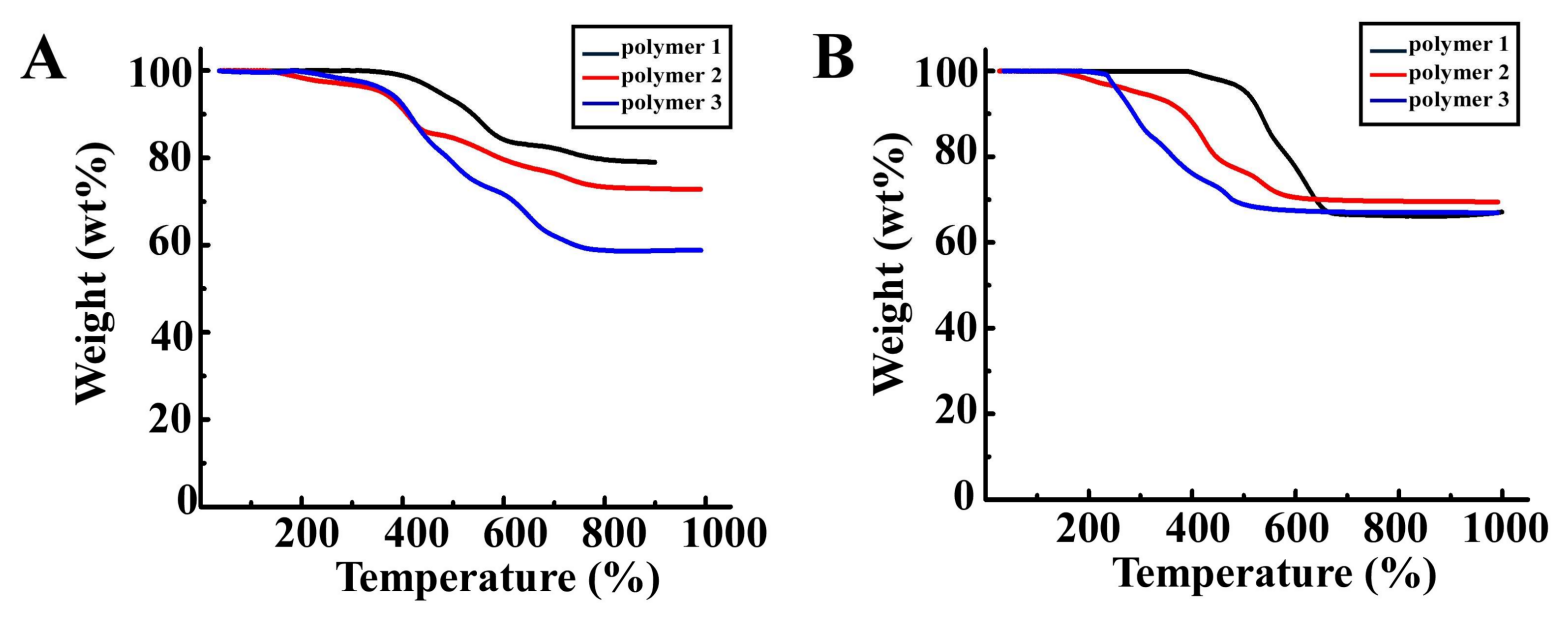
TGA plots of the polymer **1**, polymer **2** and polymer **3** under the N_2_ (**A**) and air (**B**) atmosphere respectively.

As expected, TGA indicated a very good thermal performance with 5% weight loss temperature (TD_5_) at 473 °C in nitrogen. The char value is around 80% even up to 900 °C (Figure 3A). In comparison, the TD_5_ for PDMS is only around 370 °C (in nitrogen) with a char value of below 40% at around 550 °C [44]. Apparently, polymer **1**, with an all-Si–O backbone, performs much better than regular PDMS elastomers during aging. The role of POSSs may be explained by the facilitated formation of protective silicon oxide layer on the surface during heating, and therefore reduce the weight loss during aging. The presence of phenyl groups in polymer **1** is also beneficial, since they may help to form dense carbon layer upon heat, and this then adds up another mechanism of protection [45,46].

Interestingly, the TD_5_ value is 503 °C in air (Figure 3B), presumably due to the accelerated formation of silicon oxide layer in the air atmosphere at an earlier stage than in nitrogen environment. However, the char value drops significantly down to around 65% at 900 °C and this may be explained by the reduced efficiency in the formation of carbon char by the phenyl groups in air at such high temperature, and they may instead burn up with oxygen in the air to generate volatile compounds. The performance of polymer **1** during heating in the air in terms of TD_5_ and char value is consistent with the above assumption of the roles of POSSs and phenyl groups. Polymer **1** is by far one of the most thermally stable polysiloxanes ever reported [47,48,49].

The presence of phenyl groups is crucial for high thermal performance in both nitrogen and air atmosphere. As shown in Figure 3A,B, neither polymer **2** or **3** failed to maintain as good performance as polymer **1**. In nitrogen atmosphere, the TD_5_ values for polymers **2** and **3** are 360 °C and 365 °C, respectively, and they are around 100 °C lower than that of polymer **1** (473 °C). This significant difference reflects the pivot role of phenyl by forming dense carbon layers to prevent degradation. Char values up to 900 °C are then 73% and 59% respectively for polymers **2** and **3**, also smaller than that of polymer **1** (80%).

The performance of polymers **2** and **3** in air during heating are also inferior to that of polymer **1**, but in a slightly different way. In air, the TD_5_ values for polymers **2** and **3** are 294 and 259 °C, respectively, more than 200 °C lower than that of polymer **1** (503 °C). The exaggeratedly weak performance in terms of TD_5_ values are due to the high reactivity of oxygen molecules in the air towards the organic components other than phenyl groups in the polymers. The importance of the dense carbon layer formed by phenyl groups (below 500 °C) are more obvious in air than in nitrogen, since oxygen molecules react much more readily with organic groups than nitrogen molecules, and the loss of the barrier of dense carbon layer in polymers **2** and **3** lead to more weight loss in air. In fact, the performance of polymers in air is actually more related to the realistic use of fire-retardant materials than in nitrogen. The almost unchanged char values up to 900 °C, even slighter higher in polymer **2** (69%) and polymer **3** (67%), again confirms with above conclusion that all organic groups, either phenyl or 3-chloropropyl or phosphonate groups, burn up in the air at such a high temperature, and the remaining weight are mostly ascribed to the Si/O backbone, which are almost the same for polymers **1**, **2**, and **3**, with the latter two actually slightly larger. 

### 3.3. Functional Group Variety and Application in Encapsulation of Inorganic Nanoparticles

The use of diphenyldimethoxysilane instead of diphenylsilanediol in the synthesis of polymer **1** represents the significant advantage of our approach since there are broad choice of dialkoxysilanes available either commercially or through easy synthesis. In sharp contrast, the relative diol compounds used in previous approaches are mostly limited to diphenylsilanediol and silanol-terminated polydimethylsiloxane. For the demonstration of opportunities for our polymers to advance into other areas, we then changed the starting alkoxysilane to 3-chloropropylmethyldiethoxysilane, with the hope that chloropropyl group can be chemically modified subsequently for specific purposes.

**Figure 4:**
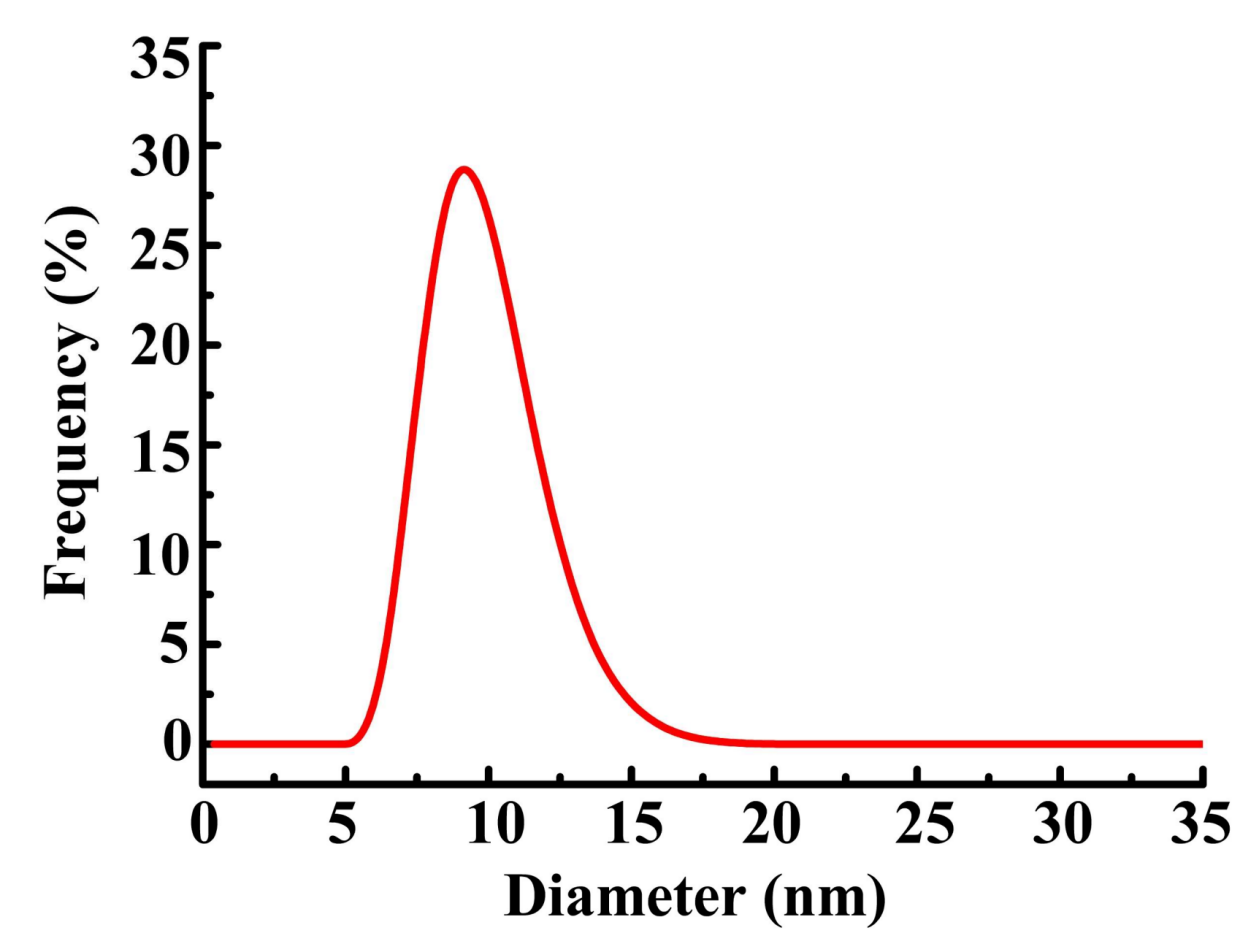
DLS of the polymer **2**, showing the diameter of around 9 nm.

The reaction between 3-chloropropylmethyldiethoxysilane and Q_8_^DMS^ was conducted similarly as the procedure for polymer **1**, and the resulting polymer **2** has a MW of 1.1 million (weight-averaged), confirming generality of this reaction (Figure S10 and Table S1). ^1^H and ^29^Si spectra (Figures S5 and S6) indicated full consumption of all Si–H and Si–OEt groups, also supported by IR (Figure S8). The size of polymer **2** (Figure 4) was close to that of polymer **1** (Figure 1D), and both were around 9 nm. The PDI of polymer **2**, however, is around 2.3, much bigger than that of polymer **1**, which is only 1.2. In fact, close inspection of the GPC curve of polymer **2** (Figure S10) revealed the bimodal nature of it while polymer **1** only exhibited a single peak (Figure S9), and this is consistent with the PDI difference. While exact cause for the broad dispersity of polymer **2** probably needs future analysis of its microstructure to distinguish different reaction pathways, the size and electronic structure of 3-chloropropyl groups is highly possible to play a role. In addition, the effect of substitution groups on the PDI was also observed in our previous report [38].

**Scheme 3:**
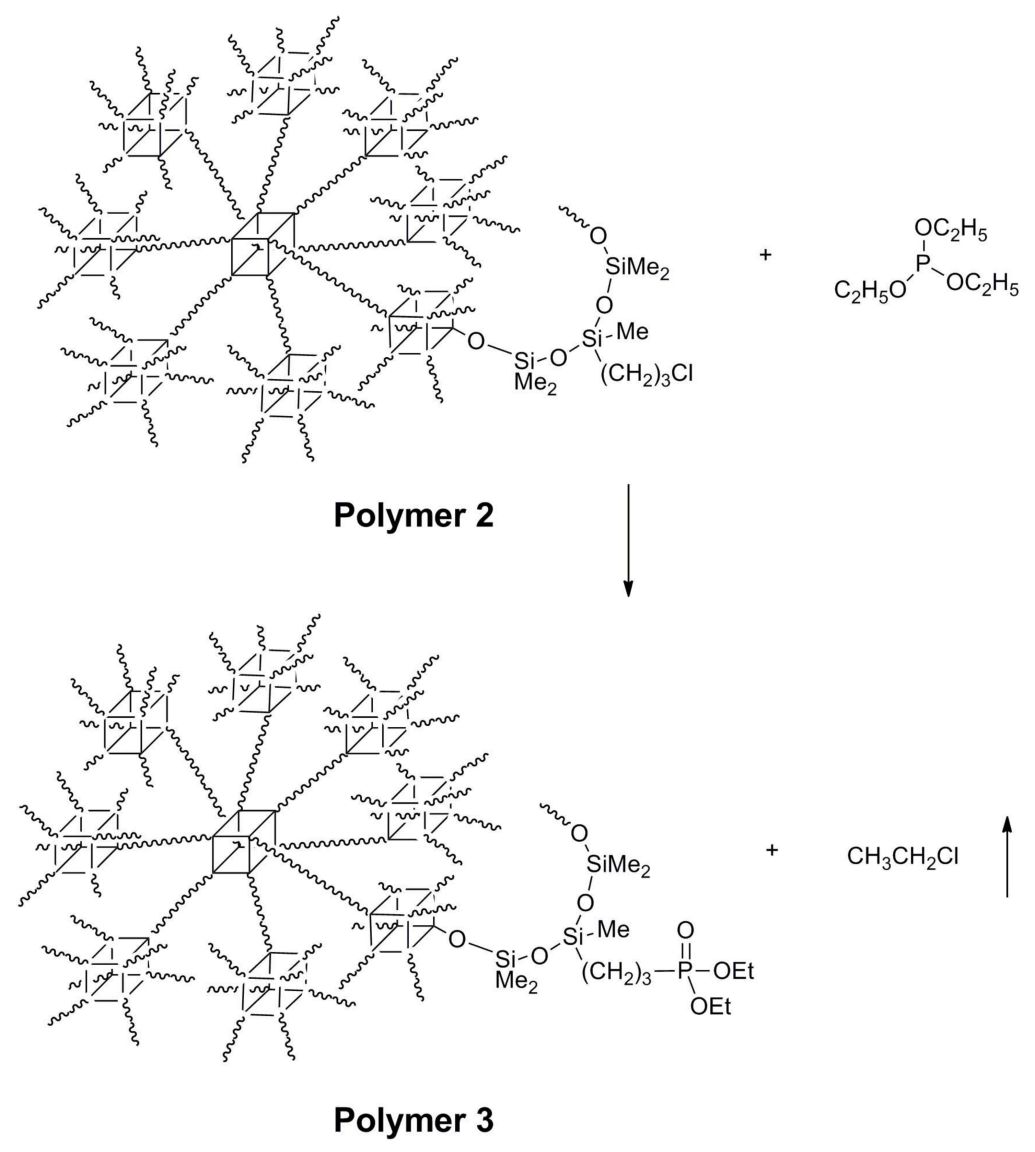
Reaction scheme to prepare polymer **3**.

**Figure 5:**
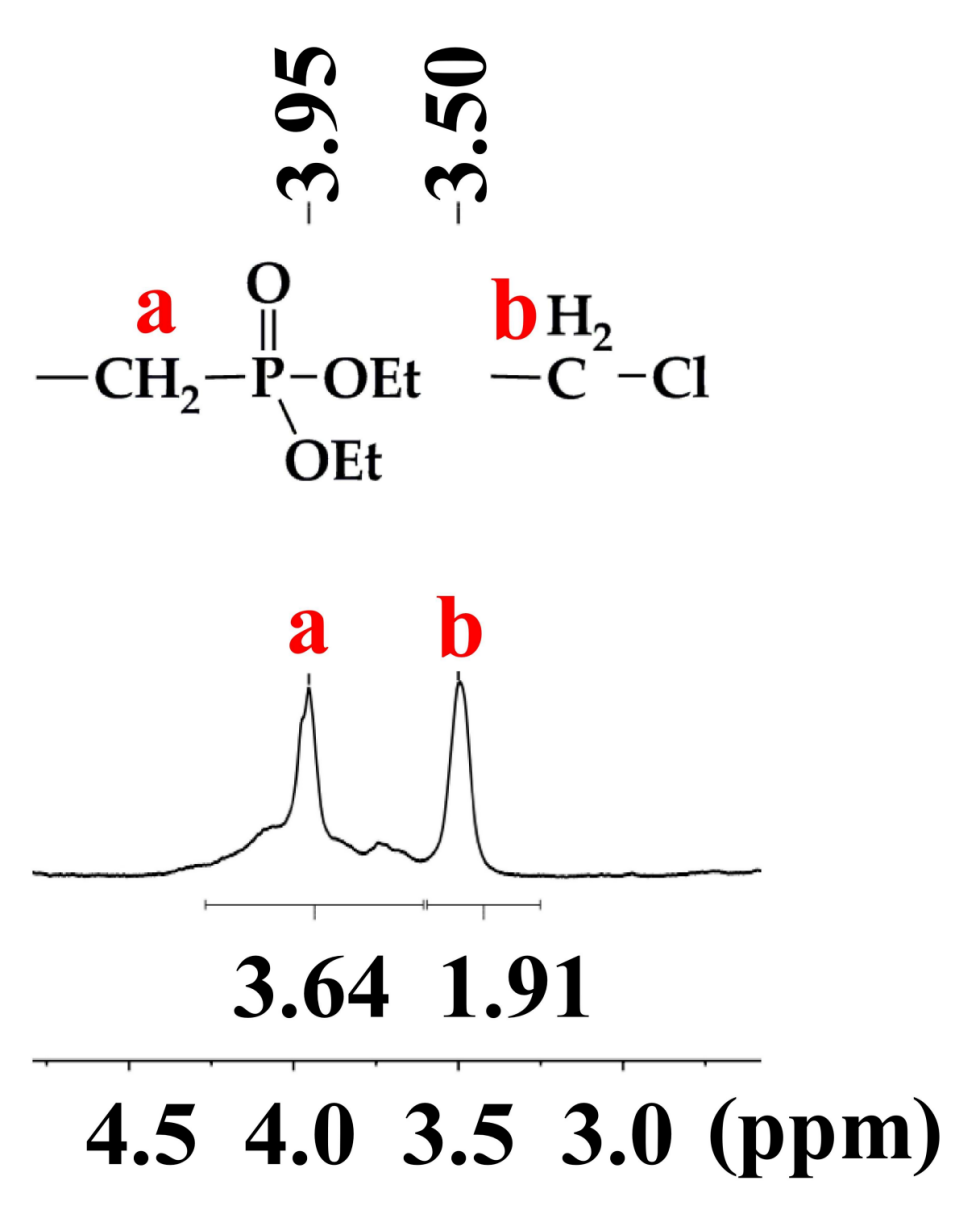
^1^H-NMR of polymer **3** and relative assignment of the peaks. The conversion of the chloropropyl groups into phosphonate-propyl groups was determined according to the integral ratio (Figure S7).

Polymer **2** was then modified further through Michaelis–Arbuzov reaction to introduce phosphonate groups (as shown in Scheme 3), since the phoshonate groups have strong affinity to a wide range of transition metals and will be helpful in preparing hybrid materials. Refluxing polymer **2** with triethylphosphite resulted in around 59% incorporation of phosphonate groups (as shown in Figure 5, detailed analysis in Figure S7), and the obtained polymer **3** dissolves in various solvents very well, but still not in methanol, probably because the polar phosphonate groups are buried within the polymer, preventing effective interaction with the methanol solvent molecules.

**Scheme 4:**
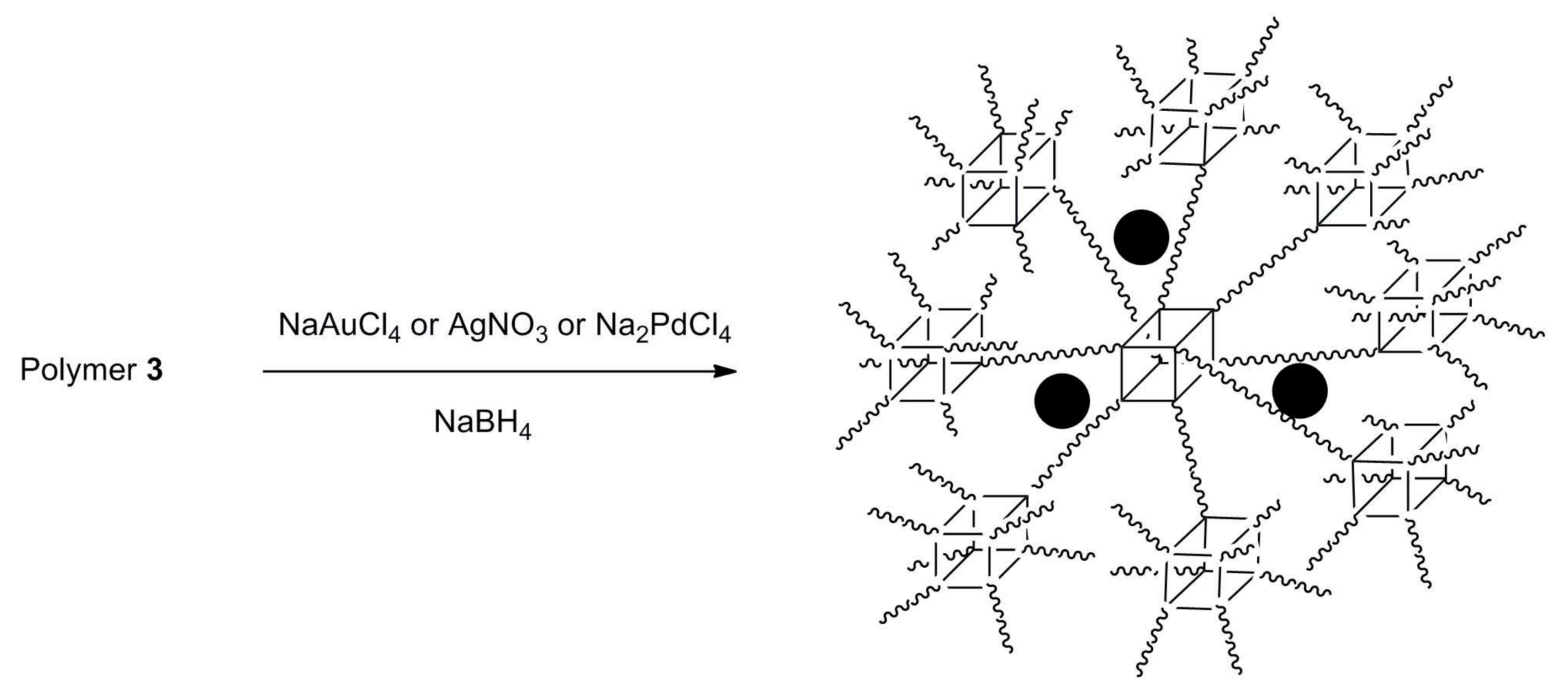
Nanoparticle (NP) encapsulation by polymer **3**.

**Figure 6:**
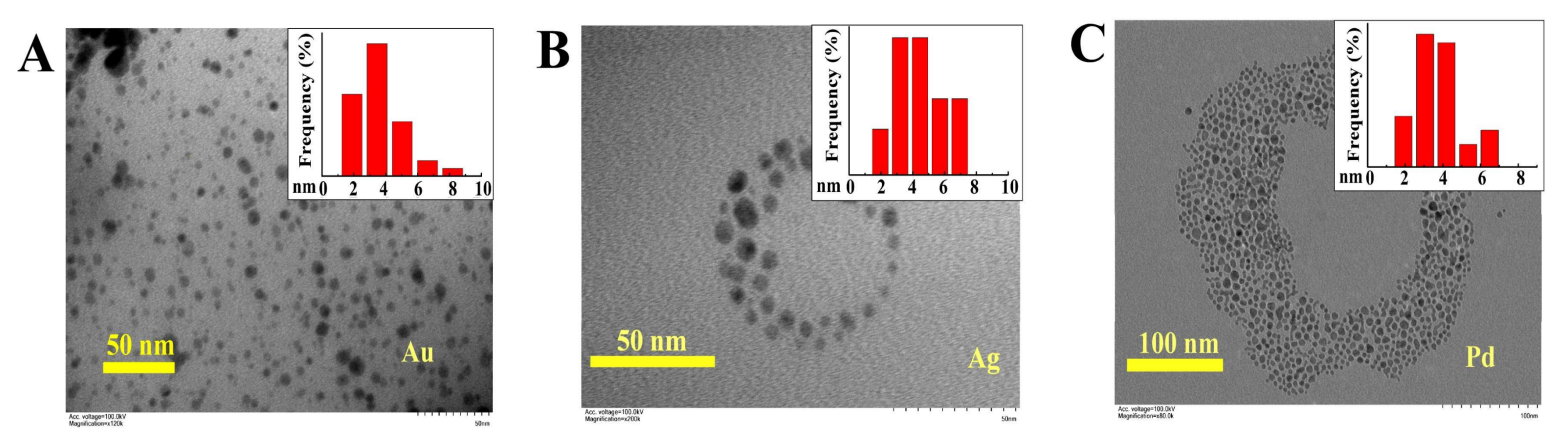
TEM images of nanoparticles of Au (**A**), Ag (**B**) and Pd (**C**) encapsulated within polymer **3**. The distribution of particle sizes is shown in the insert of each image.

Dendritic polymers, either hyperbranched ones or dendrimers, have been proved useful in stabilizing transition nanoparticles [50,51,52,53], which are of high importance in organic catalysis. The presence of rigid POSS subunits are helpful for forming internal porosity to host such nanoparticle, especially with the assistance of polar phosphonate groups introduced in polymer **3**. In fact, polymer **3** turned to be a welcome host for various transition metal nanoparticles (NPs) even without the need of hydrolysis of the phosphonate groups into relative acid. For example, reduction of NaAuCl_4_ by the NaBH_4_ in the presence of polymer **3** (Scheme 4) resulted in a clear and mauve solution without any precipitation formed. TEM analysis clearly revealed the gold NPs around 4.4 nm (Figure 6A), consistent with the size of a single polymer and therefore supporting the encapsulation of the NPs within the core of hyperbranched polymer **3**. Simply replacing the gold salts with silver or palladium ones resulted in similar encapsulated NPs by polymer **3**, as evidenced by TEM. The average sizes for Ag and Pd NPs are 5 nm and 4.3 nm, respectively (Figure 6B,C). Interestingly, for Ag and Pd NPs, in addition to the random distribution (Figure S11), there exist unexpected circular assembly of these encapsulated particles, towards which we have no clear explanation yet. We suspect that the interaction between the exterior surface of the polymer may play a role in the formation of these novel shapes. The encapsulation of inorganic nanoparticles by polymer **1** adds up the library of polysiloxanes-stabilized nanoparticles [54,55,56]. Overall, the accommodation of different transition metal NPs is very attracting since they may find application in new catalysis reactions. 

Compared to traditional polysiloxanes, which are mostly made from condensation reaction from dichlorosilane or ring opening reaction from cyclosiloxanes, our polymers present unique characters in terms of both high molecular weight and exchangeable functional groups. While traditional polysiloxanes are mostly limited to methyl version due to the fact that other functional groups bigger than methyl groups on the siloxane precursors tend to induce the formation of oligomers or short polymers, the polymers presented here are obviously free from these limitation, and will find more opportunities for utilization in a wide range of areas.

## 4. Conclusions

We report in this manuscript for the first time the synthesis of POSS-cage-containing polysiloxanes with ultra-high molecular weight and structural tuneability. The great size of a single polymer around 10 nm in the hyperbranched morphology is rare. Given the possibility of introduction of various functional groups on the backbone while still maintaining high molecular weight, this series of polymers is expected to find usefulness in other areas. The introduction of phosphonate groups serves as an introductory example for possible application, and we are currently exploring further in this direction.

## Supplementary Materials

The following are available online at http://www.mdpi.com/2073-4360/10/5/496/s1, Figure S1: ^1^H-NMR of the crystal **1**, Figure S2: ^29^Si-NMR of crystal **1**, Figure S3: ^1^H-NMR of polymer **1**, Figure S4: ^29^Si-NMR of polymer **1**, Figure S5: ^1^H-NMR of polymer **2**, Figure S6: 29Si-NMR of polymer **2**, Figure S7: ^1^H-NMR of polymer **3**, Figure S8: IR of polymer **2**, Figure S9: GPC of polymer **1**, Figure S10: GPC of polymer **2**, Figure S11: TEM images of nanoparticle coated polymers and corresponding particle size distribution, Table S1: GPC result summary of polymer **1** and polymer **2**, Table S2: Unit cell parameters of crystal **1**. The cif file of crystal **1** was deposited in CCDC database with the code of 1835605, which can be obtained free of charge from the Cambridge Crystallographic Data Center. The cif file was also provided in the supplementary materials.
